# Optimal Treatments for Severe Malaria and the Threat Posed by Artemisinin Resistance

**DOI:** 10.1093/infdis/jiy649

**Published:** 2018-12-05

**Authors:** Sam Jones, Eva Maria Hodel, Raman Sharma, Katherine Kay, Ian M Hastings

**Affiliations:** 1 Parasitology Department, Liverpool School of Tropical Medicine, United Kingdom; 2 Department of Clinical Sciences, Liverpool School of Tropical Medicine, United Kingdom

**Keywords:** *Plasmodium*, *falciparum*, malaria, artesunate, artemisinin, computer simulation, pharmacology, clinical, sequestration, pharmacokinetics

## Abstract

**Background:**

Standard treatment for severe malaria is with artesunate; patient survival in the 24 hours immediately posttreatment is the key objective. Clinical trials use clearance rates of circulating parasites as their clinical outcome, but the pathology of severe malaria is attributed primarily to noncirculating, sequestered, parasites, so there is a disconnect between existing clinical metrics and objectives.

**Methods:**

We extend existing pharmacokinetic/pharmacodynamic modeling methods to simulate the treatment of 10000 patients with severe malaria and track the pathology caused by sequestered parasites.

**Results:**

Our model recovered the clinical outcomes of existing studies (based on circulating parasites) and showed a “simplified” artesunate regimen was noninferior to the existing World Health Organization regimen across the patient population but resulted in worse outcomes in a subgroup of patients with infections clustered in early stages of the parasite life cycle. This same group of patients were extremely vulnerable to resistance emerging in parasite early ring stages.

**Conclusions:**

We quantify patient outcomes in a manner appropriate for severe malaria with a flexible framework that allows future researchers to implement their beliefs about underlying pathology. We highlight with some urgency the threat posed to treatment of severe malaria by artemisinin resistance in parasite early ring stages.

## Teaser

A new mathematical model of severe malaria calculating pathology of sequestered parasites shows the standard World Health Organization regimen outperforms a simplified artesunate regimen in patients with early stage synchronized infections. Artesunate resistance leads to worse outcomes for these patients.

**(See the Editorial Commentary by Small and Seydel on pages 1176–7.)**

*Plasmodium falciparum* is the malaria species responsible for the largest number of deaths worldwide [1] and presents clinically in 2 forms. Patients with “uncomplicated” malaria have a relatively mild fever, are conscious, and capable of taking oral drug regimens; prompt treatment of uncomplicated malaria is associated with low mortality [2]. Patients with “severe” malaria present with 1, or a combination, of 4 syndromes: severe anemia, respiratory distress, metabolic derangement, and cerebral malaria [3, 4]. Patients are treated with parenteral artesunate, which rapidly kills parasites, but resolution of pathology lags behind parasite killing; case fatality rates are high even once patients have been admitted to the formal health system (typically between 5% and 12% [2] although these have been falling to approximately 2% [5]).

A key factor responsible for severe malaria is the binding of parasitized erythrocytes (subsequently called infected red blood cells, iRBCs) to microvascular endothelium, a process known as sequestration. iRBC sequestration induces pathology through 3 main causes: (1) impairing blood flow to organs through direct physical blockage of the capillaries [6], (2) indirect blockage via host defense mechanisms such as inflammation [3, 7], and (3) physical damage to microvascular endothelium and the blood/brain barrier [8]. High case fatality rates occur, even if the drug kills parasites within sequestered iRBCs, because the molecules responsible for sequestration (eg, *P. falciparum* erythrocyte membrane protein 1 [9]) are still present on iRBC surfaces and it takes a significant amount of time for these ligands to decline sufficiently for the sequestered iRBC to detach and/or for the pathology associated with sequestration to resolve [10, 11].

Parasite clearance rates are a commonly used clinical outcome measure to compare efficacy of antimalarial treatment regimens. However, parasite clearance rates correlate poorly with disease outcome in severe malaria. Large trials comparing intramuscular artemether with quinine in African children showed more rapid parasite clearance with artemether but no difference in case fatality [12, 13]. With parenteral artesunate, parasite clearance rates are not different in patients dying from severe malaria compared to survivors (results cited in [14]). There are 2 potential explanations why parasite clearance is an unsuitable outcome measure in severe malaria: Firstly, parasite clearance rates following treatment for uncomplicated malaria appear to mainly reflect host immunity rather than drug effectiveness [15–17] so may be a poor metric of overall drug effectiveness. Secondly, parasite clearance rates are measured on circulating parasites [15] whereas noncirculating, sequestered parasites are responsible for most clinical symptoms, pathology, and deaths associated with severe malaria [3]. We developed a new model based on existing pharmacokinetic/pharmacodynamic (PK/PD) models [18, 19] (themselves based on [20–22]) to investigate 2 simple metrics reflecting the pathology of sequestered parasites in severe malaria: the maximum sequestered load posttreatment, and the area under the curve (AUC) of sequestered parasites over time posttreatment. We quantified and compared the impact of existing and proposed drug regimens on these metrics to identify rational drug dosing regimens for treatment of severe malaria. Additionally, we quantified the likely impact of artemisinin resistance in treatment of severe malaria.

## METHODS

We utilized a computer-based PK/PD model to track changes in the number of sequestered iRBCs following drug administration. The model was implemented in the statistical programming software R [23] version 3.4.1. *P. falciparum* parasites undergo a 48-hour developmental cycle in human erythrocytes with 2 main implications for pathology and treatment. Firstly, parasites initially circulate freely in blood vessels but sequester (ie, bind to capillaries) at mature stages of their intraerythrocytic cycle. Secondly, parasites differ in their sensitivity to drugs over the course of this 48-hour cycle.

**Figure 1.**
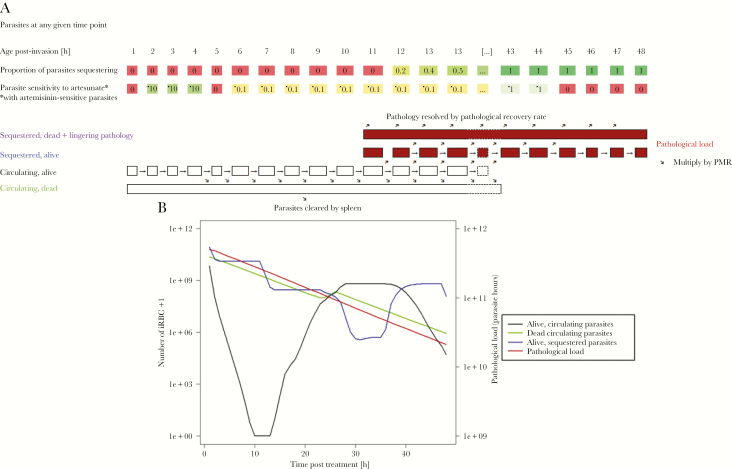
A schematic of how our model tracks parasitemia and pathology posttreatment. *A*, How the simulation tracks parasitemia and pathology. The parasite population is separated into 48 hourly “age-bins” corresponding to their developmental age within their 48-hour intraerythrocytic cycle. A certain proportion of parasites in each age-bin will be sequestered, with 0% of parasites sequestering in age-bins 1 to 11 and approximately 100% sequestering in age-bins 14–48 (the proportions given in the figure are illustrative). Parasites in age-bin 48 rupture to produce new “daughter” parasites that enter age-bin 1; the number of daughter parasites that successfully invade new erythrocytes is the parasite multiplication rate (PMR). The simulation runs in 1-hour time steps and, if drug is present, it kills parasites according to their drug sensitivity, which is given in the second row of boxes as a proportion of basal kill rate (see Supplementary Information). Parasites that survive drug action are moved forward 1 age-bin (unless they are in age-bin 48 in which case they rupture to produce daughter parasites as described above). Parasites killed by drug in the time-step have 2 fates depending on their status. Those killed in circulating stages enter a pool of “dead circulating parasites” and will eventually be removed by splenic or other host clearance mechanisms. Those parasites that are killed while sequestered are removed from the simulation but their pathology does not instantly disappear with their death, so we track their the number of dead sequestered parasites *and* the lingering pathology of these parasites (second term of Equation 1) that resolves at the user-defined “pathological recovery rate”. *B,* How this methodology is used to simulate treatment of 1 exemplar individual (recall that patients and their parasites differ in a range of important variables; see Table 1). The number of alive circulating plus dead circulating parasites can be tracked over time posttreatment. These 2 classes can be directly observed (but not distinguished) in human blood samples and their rate of clearances, usually known as “parasite clearance rate” is often used as a proxy of clinical outcome; this enables us to verify that our simulations recovered these clinical observations. Live sequestered parasites are added to the lingering effects of sequestered parasites killed in earlier stages (ie, those contributing to “post-mortem pathology”) to obtain the pathological load *L(t*) at any time point posttreatment (Equation 1). Note that the number of dead sequestered parasites and their lingering pathology are not plotted here because that line is nearly indistinguishable from total pathological load (which includes live sequestered parasites) and so only total pathological load is plotted (right *y* axis; note difference in axis scale compared to other model compartments plotted on the left *y* axis). The dynamics of *L(t*) following treatment are used to calculate our key pathology metrics that are area under the pathology curve (AUC_PL_) and the maximum parasite load (MPL). The patient displayed in this figure had sensitive parasites and was treated with the standard regimen, with PK parameters drawn from Table 1. Abbreviation: iRBC, infected red blood cell.

We assumed that severe malaria pathology is caused by a single clone (discussed in Supplementary Information) and simulated a monoclonal infection. As previously described [22], we separated the parasite population within a patient into 48 “age-bins” that each represent a 1-hour long development stage in the parasite’s 48-hour life cycle within human erythrocytes. Parasites within age-bins have differing propensities to sequester and have varying degrees of drug sensitivity. Our model tracked the number of iRBCs in each of 4 classes at any time posttreatment depending on whether the parasites are alive or dead, and whether the iRBC is circulating or sequestered: alive and circulating, alive and sequestered, dead and circulating, and dead and sequestered (see Figure 1 for illustration). Note that iRBCs classed as “dead and sequestered” are those iRBCs whose parasites have died while sequestered and are either: (1) still sequestered and causing pathology or (2) have ruptured/detached from the capillary but are still associated with continued, lingering pathology. For model specification and details, see Supplementary Information.

### Pathological Load and Pathological Recovery Rate

Severity of the malaria infection is determined by what we refer to as “pathological load,” that is the number of sequestered iRBCs (containing either living or dead parasites) physically restricting blood flow and/or eliciting patient’s immune and/or inflammatory response that may also contribute to pathology [3, 24]. It is unlikely that the iRBC immediately ruptures on death of the parasites (which would reduce physical blockage of the capillary) or that the immune/inflammatory responses immediately disappear when the parasite dies, so we assumed that pathology persists for a period after the death of the sequestered parasites. We captured this effect by defining a “pathological recovery rate,” *r*, which is the rate at which the pathology caused by sequestered iRBCs disappears with time following the death of the parasite. As will be discussed later, there are no clinical estimates of this “recovery rate” so our strategy was to quantify the impact of dosing regimen and artemisinin resistance across a range of values of recovery rate to test whether our results were dependent on assumed values for recovery rate (we show later that they were not). We varied the “recovery rate” *r* in the simulations by altering its half-life (Table 1), which is the time it takes pathology caused by dead sequestered parasites to reduce by half. We assumed that parasite death, with consequent rupturing of the iRBC or reduction of binding ligands (allowing iRBCs to detach from blood vessel walls), was essential to allow the start of pathological recovery, hence sequestered iRBCs with living parasites were not subject to the pathological recovery rate. We quantified the pathological load *L*(*t*) at any time *t* posttreatment as the sum of the current number of sequestered iRBCs with living parasites *α*(*t*) and the lingering pathological effects of once-sequestered iRBC whose parasites were killed in the current or previous time periods, *β*(*i*), that is

$$L(t)=\alpha (t)+\sum_{i=1}^{t}\beta (i)e^{-\left(t-i\right)r}$$ (1)

**Table 1.** Parameter Values Used in the Simulations^a^

Parameter	Unit	Abbreviation	Range	Format	Distribution	Justification
Initial parasite number		*P*_0_	10^*x*^, where (x∈ℝ|10<x<12)	Double	Uniform	[25, 27]
Mean of initial age-bin distribution	[h]	Mean	*x* + 0.5, where (x∈ℕ|0≤x≤47)	Integer	Triangular with mode = 10	[25, 26], Supplementary Information
Standard deviation of initial age-bin distribution	[h]	SD	*x*, where (x∈ℕ|2≤x≤4)	Integer	Uniform	[27], Supplementary Information
Parasite multiplication rate		PMR	*x*, where (x∈ℕ|1≤x≤10)	Integer	Triangular with mode = 1	[26, 27]
Pathological recovery rate half-life	[h^−1^]	*r* = ln(2)/*x*	*x*, where (x∈ℕ|4≤x≤12)	Integer	Uniform	
Splenic clearance rate half-life	[h^−1^]	*u* = ln(2)/*x*	*x*, where mean = 2.7 and CV = 0.3	Double	Normal	[28, 29]
Half-maximum inhibitory concentrations AS	[mg/L]	IC50_AS_	*x*, where mean = 0.0016 and CV = 0.86	Double	Log-normal	[20]
Half-maximum inhibitory concentrations DHA	[mg/L]	IC50_DHA_	*x*, where mean = 0.009 and CV = 1.17	Double	Log-normal	[20]
Maximal rate of drug killing	[h^−1^]	*V*_*max*_	*x*, where mean = 1.78 and CV = 0.1	Double	Normal	[20, 22]
Slope factor		*n*	*x*, where mean = 4 and CV = 0.3	Double	Normal	[20]

Abbreviations: AS, artesunate; CV, coefficient of variation; DHA, dihydroartemisinin.

^a^Not including volume of distribution (*V*_*d*_) / clearance (*Cl*). See Supplementary information for discussion of those parameters.

We used 2 metrics to analyze treatment regimens and resistance: (1) maximum pathological load (MPL), the maximum value of *L(t*) occurring during a defined time period posttreatment, and (2) the area under the pathological load curve (AUC_PL_) during a defined time period posttreatment, that is the total pathology in that period. For example, the AUC_PL_ in the period 0 to 24 hours posttreatment is:

$$AUC_{PL}=\sum_{t=1}^{24}L\left(t\right)$$ (2)

### Simulating Patient Treatment Cohorts

We simulated a cohort of 10000 patients who had parasitological, pharmacological, and patient-specific parameters drawn from the distributions given in Table 1. Individual patient profiles allowed individual PK/PD variation to be incorporated to generate individual patient posttreatment parasite clearance dynamics (Supplementary Information). Each patient was simulated 3 times under different scenarios: once for drug-sensitive parasites treated by the standard World Health Organization (WHO) regimen (2.4 mg/kg artesunate twice a day in the first 24 hours), once for sensitive parasites treated with the simplified regimen (4 mg/kg artesunate once a day, as proposed by Kremsner et al [30]), and once for artemisinin-resistant parasites treated by the standard WHO regimen. This allowed us to compare the 2 dosing regimens (“standard” vs “simplified”) and the impact of resistance (“sensitive” vs “resistant”) *in each patient*. Follow-up time was 48 hours after drug administration; this reflected a whole parasite life cycle within an iRBC but, more importantly, covers the period posttreatment where a patient is most likely to die [31, 32].

### Sensitivity Analysis

We conducted partial rank correlation coefficient (PRCC) using Spearman ρ to establish the strength of the relationship between model parameters and dependent variables (ie, the pathology metrics AUC_PL_ and MPL).

All parameters are quantitative so can enter the PRCC without modification. The exception is mean age-bin which, although numeric, has a “circular” scale, age-bin 1 being adjacent to age-bin 48, due to parasites from ruptured iRBCs (at hour 48) reinvading to restart the asexual life cycle. The mean age-bin variable was therefore split into either 5 or 3 ordinal classes (depending on whether parasites were hypersensitive or resistant to artemisinin), as described in Supplementary Information.

The following parameters were included in the PRCC analysis:

• duration of artesunate killing posttreatment; this captures all the PK/PD parameters in Table 1 except maximal artesunate kill rate
• maximal rate of artesunate killing (*V*_*max*_)
• initial mean age-bin as a categorical variable (see above)
• variation of initial age-bin distribution (measured as the standard deviation (SD) around the mean)
• initial parasite number
• parasite multiplication rate (PMR)
• half-life of the ‘pathological recovery rate’ (*r*).

The splenic clearance rate was not included in the analysis as it has no impact on sequestered iRBC based pathology.

## RESULTS

**Figure 2.**
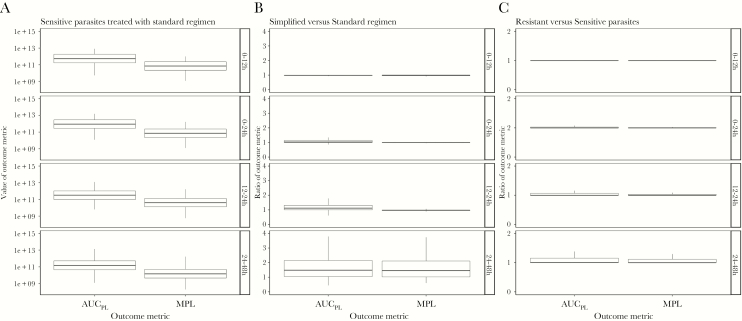
Values of area under the pathology curve (AUC _PL_) and maximum parasite load (MPL) obtained for each of 3 model scenarios across 4 time periods posttreatment: 0–12 hours, 0–24 hours, 12–24 hours, and 24–48 hours. *A*, The “baseline scenario” when artemisinin-sensitive parasites are treated with the standard regimen. *B*, A comparison of the simplified versus standard regimen (values >1 show the standard regimen is superior). *C*, A comparison of the standard regimen when used to treat resistant versus sensitive parasites (values >1 show that sensitive parasites produce better outcomes).

Our model calculated pathological load and returns 2 outcome metrics: AUC_PL_ and MPL. Figure 2 shows the values of these metrics for 3 model scenarios: patients with sensitive parasites treated with the standard WHO regimen, a comparison of the ratios of AUC_PL_ and MPL for treatment with simplified regimen versus standard regimen, and the impact or artemisinin resistance on outcomes following treatment with standard WHO regimen.

Ratios of outcome metrics are calculated as simplified regimens scaled by standard regimen and as resistant parasites scaled by sensitive parasites. High metrics are deleterious, thus ratios of >1 indicate worse prognosis associated with the simplified or resistant parasites. These ratios quantify the impact, for example a ratio of 5 for resistant versus sensitive parasites indicates pathological metrics are 5 times higher when treating resistant parasites. We investigated 4 time periods posttreatment: 0–12 hours, 0–24 hours, 12–24 hours, and 24–48 hours.

### Consistency of Model Outputs with Existing Field Data

Our model calculated parasite reduction ratios (PRR) from circulating parasite numbers (Supplementary Information). The clinical endpoint of the trials by Kremsner and colleagues was the proportion of patients in each arm whose PRR at 24 hours (PRR_24_) was >99% [30], reported as 79% and 78% for the 5-dose standard and the 3-dose simplified regimen, respectively. When calibrated with PK parameters from Kremsner’s study [30], our results were consistent with these clinical observations, that is our model predicted 78% and 74% for the standard and simplified regimen with hypersensitive parasites, respectively (Supplementary Table 3). However, the results we present below are calibrated using PK parameters from Hendriksen et al [33] (see Supplementary Information for justification), with which we observed lower values of 70% and 62% of patients with PRR_24_ >99% for the standard and simplified intramuscular regimens, respectively.

Hendriksen et al [33] do not report the percentage of patients with PRR_24_ >99% in their study, so we could not simultaneously compare the findings of our simulation with the findings of Kremsner et al [30] and Hendriksen et al [33]. However, Hendriksen et al [33] reported the population geometric mean of the fractional reduction in parasite counts at 24 hours as 96% (95% confidence interval [CI], 94%–98%,) following treatment with the standard regimen. The population geometric mean obtained for the reduction in parasite counts at 24 hours (ie, PRR_24_) in our simulation using parameters from Hendriksen et al [33] was >99%.

**Figure 3.**
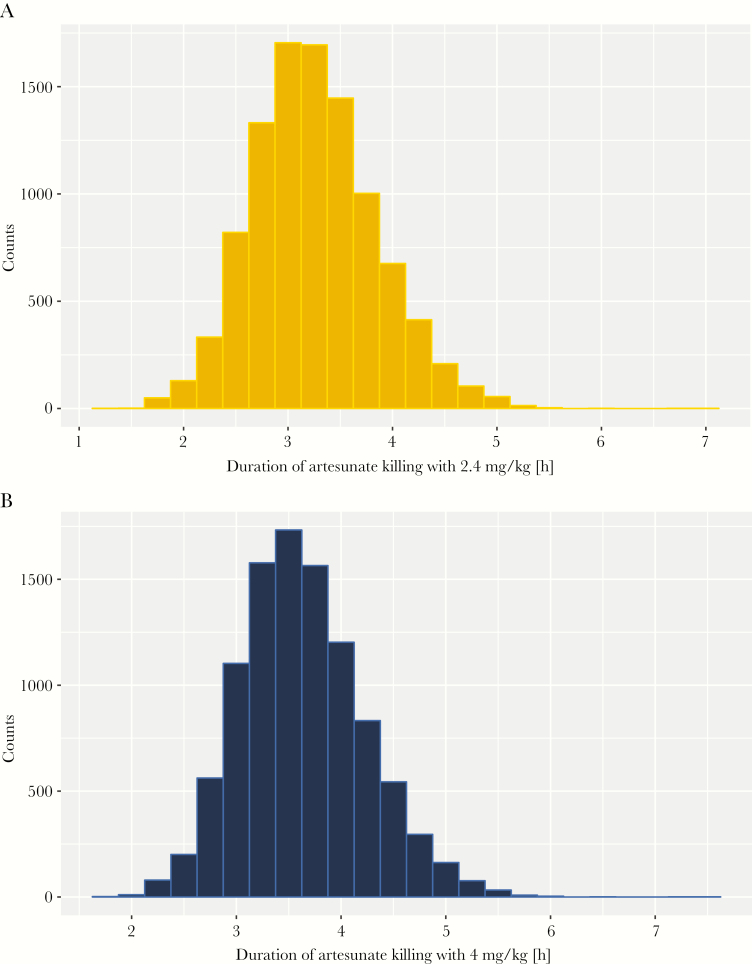
Distribution of artesunate killing duration. Data for 10000 patients following treatment with a single dose of artesunate of either 2.4 mg/kg (*A*) or 4 mg/kg (*B*); note the duration includes that of the active metabolite dihydroartemisinin. This distribution was obtained using parameters from Hendriksen et al [33].

The general accepted value for PRR_48_ following artemisinin treatment is 10^–4^ [34], which is very close to the value obtained here: for the standard regimen, using the artesunate killing duration derived from Hendriksen’s PK parameters (Figure 3) we obtained a mean PRR_48_ of 5.18^−5^ (see Supplementary Information for a nuanced discussion of PK parameters).

### Standard Regimen Treatment of Artemisinin-Sensitive Parasites

**Figure 4.**
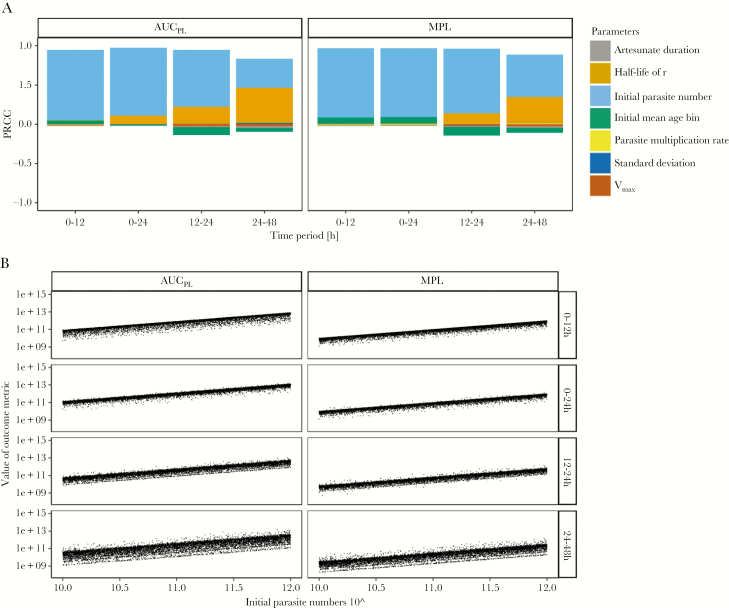
Analysis of the baseline scenario. The impact of underlying factors on the standard World Health Organization regimen used to treat patients with artemisinin-sensitive parasites. *A*, Partial rank correlation coefficients (PRCC) using Spearman *ρ* of model parameters on values of area under the pathology curve (AUC_PL_) and maximum pathological load (MPL) obtained from a population. *B*, Values of AUC_PL_ and MPL are plotted against the most highly correlated parameter, that is initial parasite number, for 4 time periods posttreatment.

We simulated treatment of drug-sensitive parasites with the standard regimen and identified the key drivers of pathology by calculating which parameters were most correlated with AUC_PL_ and MPL (Figure 4;  Supplementary Table 7). The most highly correlated parameter for both metrics was the initial parasite number: large positive PRCCs (between 0.88 and 0.98) were observed with associated *P* values ≤.001 at all time periods. The half-life of the recovery rate *r* had PRCC of 0.46 for AUC_PL_ and 0.34 for MPL in the 24 to 48-hour time period (*P* values ≤.001), but PRCC of <0.3 in earlier time periods. All other parameters had PRCC values of <0.3, indicating that outcome metrics were not highly correlated as per accepted statistical criteria [35]. All other model parameters had negligible correlation. The most likely explanation is that such a large proportion of parasites are killed by artesunate that small differences in the number killed are negligible compared to the initial parasite number and pathological recovery rate.

### Comparison of Simplified and Standard Regimen

We evaluated alternative treatment regimens on artemisinin-sensitive parasites. These results are presented as ratios of AUC_PL_ and MPL. The simplified regimen had a slightly higher median ratio in 0–24 hours of 1.03; MPL was 1. At 24–48 hours, higher medians of 1.49 and 1.45 for AUC_PL_ and MPL, respectively, were observed (Figure 2; Supplementary Table 4).

**Figure 5.**
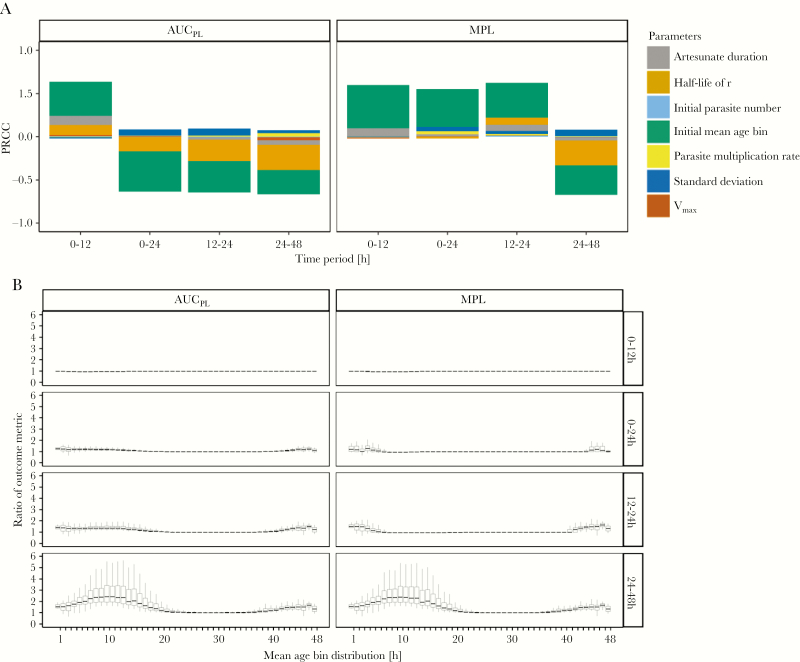
Evaluation of alternative drug treatment regimens. Comparison of the simplified versus World Health Organization standard regimen for treatment of artemisinin-sensitive parasites; ratios of >1 indicate the simplified regimen produces worse outcome metrics. *A*, Partial rank correlation coefficients (PRCC) using Spearman *ρ* of model parameters on the ratios of area under the pathology curve (AUC_PL_) and maximum pathological load (MPL). *B*, Ratios of AUC_PL_ and MPL are plotted against the most highly correlated parameter (initial mean age-bin), for 4 time periods posttreatment.

Parameter analysis with PRCC (Supplementary Table 8) revealed that patients whose initial infections were in either very late or very early initial mean age-bins (Figure 5, lower panel) will have worse outcomes with the simplified regimen. This occurred because parasites in these stages are largely insensitive to artesunate at first treatment, and the simplified regimen lacks the second dose, 12 hours later, of the standard regimen that would effectively target these parasites that had matured into more artemisinin sensitive age-bins.

The half-life of the recovery rate *r* had a moderate correlation with outputs in the 12 to 24-hour and 24 to 48-hour periods, indicating that assumption of slower recovery made the simplified regimen perform relatively better (Supplementary Figure 5). We are confident this parameter does not affect the validity of our results; for complete discussion see Supplementary Information. No other parameters have notable correlation with sequestration-based pathology when comparing regimens.

We repeated this analysis to compare regimens (ie, WHO standard vs simplified) when treating artemisinin-resistant parasites. Differences between regimens were extremely similar to those shown in Figure 5 and are displayed in Supplementary Figure 7 and Supplementary Table 9.

### The Impact of Artemisinin Resistance on Treatment by the Standard Regimen

Unsurprisingly, ratios of AUC_PL_ and MPL when comparing resistant and sensitive parasites are never less than 1 (Figure 2), that is under no circumstance did patients have a better outcome when parasites are resistant. Differences in median values (Figure 2; Supplementary Table 4) were extremely small.

**Figure 6.**
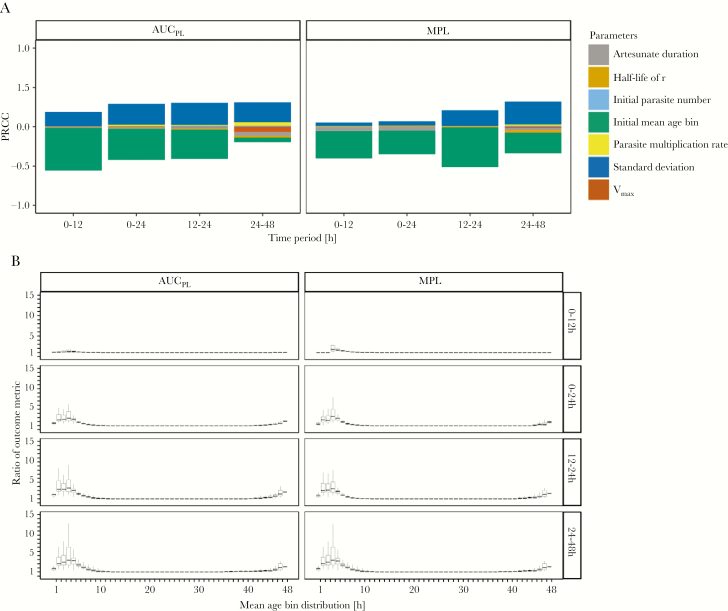
Analysis of the impact of artemisinin resistance. The effectiveness of the World Health Organization standard regimen used to treat resistant versus sensitive parasites; ratios of >1 indicate that resistant parasites have worse outcome metrics. *A*, Partial rank correlation coefficients (PRCC) using Spearman *ρ* of model parameters on the ratios of area under the pathology curve (AUC_PL_) and maximum pathological load (MPL). *B*, Ratios of AUC_PL_ and MPL are plotted against the most highly correlated parameter (mean age-bin), for 4 time periods posttreatment.

We carried out PRRC analysis (Supplementary Table 10) to investigate whether this small difference obscured the presence of a vulnerable subgroup of patients. This appeared to be the case: patients whose infections are clustered in the early age-bins at time of treatment had pathological outcomes that were significantly worse in the presence of resistance (Figure 6).

In these early age-bins, ratios for AUC_PL_ and MPL are as high as 5 in the 0 to 24-hour period (comparisons based on the upper quartile value). This occurs because artesunate presence posttreatment largely coincides with parasites in age-bins insensitive to artesunate through resistance, rendering the initial dose nearly or completely ineffective.

SD of the initial mean age-bin had a positive correlation with the ratio (indicating that resistant parasites had worse outcomes as SD increased). This occurred because higher SD “nudged” parts of the age-bin distribution into (or out of) resistant age-bins (ie, the contiguous bin 45–48 and 1–5 where killing is absent). PRCC analysis showed no other parameter had a PRCC value of >0.01, suggesting the initial mean age-bin (and, to a lesser extent, its SD) are the sole determinants of whether a patient’s outcome will be worse in the presence of resistance.

## DISCUSSION

We established a PK/PD modeling methodology capable of investigating the treatment of severe malaria. Kremsner et al [36] recognized the clinical necessity of this, and noted that “for the first time, we [ie, Kremsner et al] are assessing artesunate using similar pharmacokinetic and dynamic approaches”. Parasite clearance is likely to be a poor measure of regimen effectiveness (and, by extension, clinical outcome) in severe malaria where pathology is due to sequestered parasites. The effects of alternative regimens and the impact of drug resistance can only be investigated by traditional clinical outcomes using large-scale clinical trials, so pharmacological modeling of the type proposed here is essential to help generate the evidence base for rational treatment design. Our pathological modeling was highly flexible (discussed in Supplementary Information) and, of necessity, reflected the limitations in our understanding of pathology, for example how rapidly pathology is resolved following parasite death and whether pathology depends on maximal sequestered load (measured as MPL) or on total exposure (measured as AUC_PL_). An interesting, highly important result is that the key quantitative assumption made in the analysis, the rate of resolution of pathology (measured as the half-life of *r*), had little effect on our conclusions when comparing alternative regimens or the impact of resistance (Supplementary Information) implying that the pathological model is a robust to assumptions made in this comparative investigation. Importantly, while circulating parasite loads do not reflect the pathology of severe malaria they are currently the regular endpoint of choice in severe malaria trials, including those undertaken by Kremsner et al [30, 37]; our model was able to reproduce the clinical outcomes reported in [30, 33] (when appropriately parameterized), and recover expected PRR_48_, so we are confident it is reflective of in vivo scenarios (Supplementary Information).

Kremsner and colleagues [30, 37] concluded that their simplified regimen was noninferior to the standard WHO regimen and possessed operational advantages due to less frequent drug administration [30, 37]. This work was influential and initiated a wider debate about the best drug regimen(s) to treat severe malaria [14, 36, 38] to which our study can contribute. Comparison of the 0 to 24-hour and 12 to 24-hour period was used to compare the effects of the initial, larger dose of the simplified regimen against the additional dose at 12 hours with the standard regimen. The standard regimen produced slightly lower median AUC_PL_ within the first 24 hours posttreatment (Figure 2; Supplementary Table 4). This difference was greater in the 24 to 48-hour period, but the majority of pathological load occurred within the first 24 hours as artesunate rapidly kills parasites: AUC_PL_ in the 24 to 48-hour period is, on average, between 20% and 30% that of AUC_PL_ in the 0 to 24-hour period (data not shown). The first 24 hours are critical for patient survival [31], so outcome metrics at 24–48 hours may have little relevance in choosing between regimens. However, the simplified regimen performed much worse in the subgroup of patients with very late or very early initial mean age-bins. Based on these results, we are dubious about recommending use of the simplified regimen but add an important rider to this. Kremsner et al never claimed this simplified regimen would be superior, but argued that any inferiority, if it exists, would be within acceptable margins. We leave it to clinically qualified personnel to judge whether 50% in some subgroups is within an acceptable margin of inferiority, especially given our inability to directly link our pathological outcomes with the likelihood of mortality.

We assessed the impact of artemisinin resistance on treatment of severe malaria, that is the extent to which resistance increased MPL and AUC_PL_. Resistance prevents drug killing in age-bins 2–4 (these bins are otherwise hypersensitive) resulting in no killing for a contiguous 8-hour period in resistant parasites (ie, age-bins 45 to 5). Our results show the initial mean age-bin and its SD are the only parameters that distinguish outcomes between sensitive and resistance parasites (Figure 6). We argued previously [39] that artemisinin resistance would have a negligible impact on eventual cure rates in uncomplicated malaria (provided there was no resistance to partner drugs) but artemisinin resistance clearly poses a much larger threat to treatment of severe malaria than it does to uncomplicated malaria. Although differences between sensitive and resistant parasites across the entire population are minor (Figure 2; Supplementary Table 4), there is an extremely vulnerable subgroup of patients whose infections at the time of treatment are clustered in very late or very early age-bins (ie, where parasites are resistant in our model; Figure 6).

Note that we specifically model relatively tightly synchronized parasite distributions (Supplementary Information); if distributions were to become less tightly synchronized the vulnerability of patients with early initial mean age-bins decreases and both the difference between regimens and the impact of resistance reduces.

We present a highly adaptable methodology for PK/PD modeling of treatment of severe malaria that was able to recover key clinical observations (based on circulating parasite numbers), and, with novel metrics, used to investigate the pathology of severe malaria. Our model showed that while on a population level a simplified artesunate regimen is noninferior to the standard WHO regimen, outcomes in a subgroup of patients with infections grouped in late or early initial mean age-bins are notably worse with the simplified regimen. The emergence of artemisinin resistance in early ring stages poses a significant threat to this same group of patients. Neither of these results are particularly obvious from summary statistics of the population and so subgroup analysis is particularly important in devising treatment strategies for severe malaria.

## Supplementary Data

Supplementary materials are available at *The Journal of Infectious Diseases* online. Consisting of data provided by the authors to benefit the reader, the posted materials are not copyedited and are the sole responsibility of the authors, so questions or comments should be addressed to the corresponding author.

Supplementary Material

Supplementary Table S1

Supplementary Table S2

Supplementary Table S3

Supplementary Table S4

Supplementary Table S5

Supplementary Table S6

Supplementary Table S7

Supplementary Table S8

Supplementary Table S9

Supplementary Table S10

Supplementary Figure S1

Supplementary Figure S2

Supplementary Figure S3

Supplementary Figure S4

Supplementary Figure S5

Supplementary Figure S6

Supplementary Figure S7

## Notes

***Acknowledgment.*** We thank 2 anonymous reviewers for their helpful comments and critique.

***Financial support.*** This work was supported by the UK Medical Research Council (grant numbers G1100522 and MR/L022508/1); the Bill and Melinda Gates Foundation (grant number1032350); and the Malaria Modeling Consortium (grant number UWSC9757).

***Potential conflicts of interest.*** All authors: No reported conflicts of interest. All authors have submitted the ICMJE Form for Disclosure of Potential Conflicts of Interest. Conflicts that the editors consider relevant to the content of the manuscript have been disclosed.
