# Supplementary material for: Optimal Treatments for Severe Malaria and the Threat Posed by Artemisinin Resistance
Source: J Infect Dis. 2018 Dec 5;219(8):1243–53. doi: 10.1093/infdis/jiy649 (PMC6452316; doi:10.1093/infdis/jiy649)
Supplement: Supplementary Material [file jiy649_suppl_supplementary_material.docx]

Supplementary Information.

Table of Contents

[Model specification: Age-bins, sequestration and drug killing 1](#_Toc526239742)

[Measuring parasite clearance: Parasite reduction ratios 7](#_Toc526239743)

[Simulating treatment of adults 8](#_Toc526239744)

[Metrics of severe malaria pathology 9](#_Toc526239745)

[Impact of the recovery rate *r* (and its half-life) 11](#_Toc526239746)

[Choice of Standard Deviation (SD) of the initial mean age bin and modelling uniform age bin distributions. 15](#_Toc526239747)

[Supplementary information references: 17](#_Toc526239748)

# Model specification: Age-bins, sequestration and drug killing

Note that we explicitly assume only a single clone is responsible for sequestration-based pathology, consistent with existing research [1, 2]. Modelling a mixed infection with our model, should a reader desire, is straightforward: Two or more clones can be simultaneously tracked in the same individual by running one simulation for each clone. Pathology is additive (main text) so total pathology is the sum of the individual clonal pathologies. Note that PK parameters are patient-specific so need to be held constant but PD parameters such as initial mean age-bin and SD can be explicitly varied for each clone.

Developmental age-bins are enumerated chronologically: Bin 1 represents an infected red blood cell (iRBC) in the first hour following parasite invasion, and bin 48 is the final age-bin. Parasite development ends after bin 48 with the rupturing of the iRBC to release merozoites that re-invade RBCs and re-enter age-bin 1. The intra-host model tracks the number of parasites in each of the 48 age-bins at each time point post-treatment. Specifically, the number of alive iRBCs (both circulating and sequestered) at each time-point in each bin was tracked and used to calculate the number of alive iRBCs in the next age bin at the next time-point as:

  Equation S1

where represents the number of alive iRBCs in age-bin *b*+1 at time *t*+1 post-treatment, which depended on the iRBCs in the current age-bin and time period and the proportion which survived drug treatment in the bin at time where is the drug-specific killing rate for that age bin, i.e. the proportion of parasites killed in age bin *b* at time *t* (see below). The only exception is for age-bin 1 which reflected parasites released from iRBCs at age-bin 48 i.e.

 Equation S2

where PMR is the parasite multiplication rate, i.e. the number of merozoites released from a schizont that successful infect a new erythrocyte (**Table 1**, main text).

Sequestration was incorporated by following, for convenience, the assumption of Saralamba *et al.* [3], i.e. that parasites begin to leave circulation and sequester in/after age-bin 11, with a half-life of z=3 hours, such that 50% of parasites are sequestered when reaching age-bin 14. Consequently, the proportion of parasites in age-bin, *b* that remain in the circulation, *P_c_ ,* is

$P_{C}=2^{\frac{11-b}{z}}$ (for 12 ≤ b ≤ 48) Equation S3

and

$P_{C}=0$ (for 1 ≤ b ≤ 11) Equation S4

The number of alive iRBCs (Equation S1, Equation S2) in each stage can then be multiplied by the proportion of iRBCs circulating in each stage (Equation S3, Equation S4) to calculate the number of parasites in the alive circulating and alive sequestered compartments (this is done before incorporating drug killing in that stage).

Our first objective was to model the likely extent of pathology caused by sequestered iRBCs in patients treated with two alternative regimens of artesunate treatment i.e. The “Standard” WHO recommended regimen [4] of 2.4 mg/kg i.m., given twice in the first day and daily thereafter, i.e. at 0, 12, 24, and 48 hours (referred to by Kremsner *et al.* [5] as the “five dose regimen” - an additional dose is given at 72 hours)

The “Simplified” regimen as proposed by Kremsner *et al.* [5] (and referred to by them as the “three dose regimen”) consisting of larger doses of 4 mg/kg i.m. given once each day i.e. at 0, 24 and 48 hours.

We parameterised our simulation for artesunate treatment which is the World Health Organization (WHO)-recommended drug for the treatment of severe malaria [6]. Artesunate is eliminated so rapidly that it decays from concentrations generating maximal killing rates to physiologically negligible concentrations very rapidly; this means that it can be regarded as being either present (and killing at maximum rate) or absent [7]. The PK parameters describing paediatric artesunate treatment were drawn from Hendriksen *et al*. [8].

We initially utilized the PK parameters provided by Kremsner *et al.* [5] (**S1 Table**) to generate artemisinin killing durations for our patient population, but found the distribution to be an atypical shape to that which would usually be expected (**S1 Figure**) – this is likely because we were unable to model the correlation between Vd and CL using the parameters provided by Kremsner *et al.* [5]. Notably, however, use of their PK parameters and this distribution did allow for the recovery of Kremsner’s clinical observations (Figure 5 of [5]), see main text results.

As mentioned above, the atypical distribution of artemisinin killing durations may be due to the correlation between Vd and CL but this was impossible for us to calculate using the parameters given in [5]. Thus, we utilized the PK parameters from Hendriksen *et al.* [8] who provided the random effects that allowed us to incorporate parameter correlation. Specifically, we draw our population estimates for the mean of Vd and CL from the fixed effects in Table 2 of [8] and the associated CV of these parameters from the random effects (ETA), where we calculated CV assuming they used a proportional or exponential error structure: CV = sqrt(exp(ETA)-1). We also incorporated the correlation term “*ηCL/F ~ ηV/F”* listed under random effects and assumed that Vd and CL were correlated in our simulation. The PK values we used in our simulations are thus summarized in **S2 Table**.

The resulting distribution using Hendriksen *et al*. [8] parameters (Figure 2, main text) was appropriately shaped. The clinical findings obtained using Kremsner *et al.* [5] and Hendriksen *et al.* [8] parameterizations in our simulations are shown in S3 Table.

In our model, the duration of artesunate killing was determined as follows: First the kill rate-over-time profiles for artesunate and its active metabolite dihydroartemisinin (DHA) were calculated using standard equations [9, 10] based on the dose and an individual’s PK/PD parameters **(Table 1, main text**). The time at which the half-maximal rate of drug killing (*V_max_*/2) occurs for both artesunate and DHA was used to estimate the duration of killing by both; duration of artesunate killing was set to whichever was longer.

Our PK/PD model uses one-hour time steps. Thus, a patient with “extra” killing over an hour would have that “extra” killing added to the next hour, such that a patient with 2.2 hours killing would have 2 hours of killing at V_max_ and would have 0.2*V_max_ killing in the third hour. Any values of artesunate duration below 1 hour or above 12 hours (for a single dose) were deemed unrealistic and resampled. See Figure 2 for the distributions of artesunate killing duration.

Stage-specific drug sensitivity was incorporated as previously described [7] using their “hyper sensitive” profile to include findings from the rings-stage assay (described by [11]) which revealed parasites to be very sensitive to artemisinin just after their invasion of erythrocytes. Thus, our “sensitive” profile assumed a “baseline” sensitivity to artesunate in parasite age-bins 18 to 44 inclusive (Equation S7), hyper-sensitivity in age-bins 2 to 4 inclusive (Equation S5) which were ten times more sensitive than baseline, and reduced-sensitivity stages between age-bins 6 and 17 inclusive which were ten times less sensitive than baseline (Equation S6). Drug killing in other age-bins is 0. The factor *D* ^x-y^ denotes drug killing between age-bins x to y inclusive and is defined as

*D^2-4^* = Equation S5

*D*^6-17^ = Equation S6

*D^18-44^*= Equation S7

$D^{1}=D^{5}=D^{45-48}=0$ Equation S8

where *d* is the duration of the time-step (i.e. one hour in this case). The mean value of *V_max_* is 1.78 as estimated previously [12] (**Table 1, main text**).

Recent reports of artemisinin “resistance” suggest resistance is restricted to the early “hyper-sensitive” stages [13] so we investigated the impact of this resistance by setting the artesunate sensitivity of parasites in these age-bins (i.e. 2-4) to zero, while killing in other age-bins is unchanged. This “resistant” profile is therefore described as

*D*^6-17^ = Equation S9

*D^18-44^*= Equation S10

$D^{1-5}=D^{45-48}=0$  Equation S11

The mean age bin must be grouped as a categorical variable for the purposes of carrying out Partial Rank Correlation Coefficient (PRCC) analysis; each category must be given an ordinal rank. Age-bins have different sensitivity to artesunate (Equations S5-11) and so were ranked according to the amount of drug killing that occurred in that age-bin, the category with lowest killing being the lowest rank and the category with highest killing being the highest rank. The categories are shown in **S5 Table** for sensitive parasites and **S6 Table** for resistant parasites.

Note that, for sensitive parasites, while age bins 45-1 and 5 have the same (lack of) killing, 45-1 is given a lower rank as it is a longer, more continuous section without killing, so it felt prudent to separate 45-1 and 5 into different categories.

*In vivo*, fever brought on by host immunity is thought to play a role in killing parasites in cases of severe malaria. Various existing models of malaria account for this effect by preventing parasite numbers reaching unfeasibly high levels [14, 15]. We bring this regulation into our model by assuming “fever” and/or other immune mechanisms act to slow the multiplication rate of the parasites. This is the “parasite multiplication rate” (PMR) which we vary from 1 to 10 using a triangular distribution with mode =1 (Table 1). Obviously, a value of 1 indicates the parasitaemia is being controlled and held constant by host mechanisms but we thought it important to investigate situations where parasitaemia is not completely regulated (hence PMR can go up to 10). This assumption is justified in the publications we cite in Table 1. Additionally, note that PMR is not a parameter that is correlated with ratios of outcomes for either comparison of regimens or resistant / sensitive parasites (results, main text).

# Measuring parasite clearance: Parasite reduction ratios

We used the model to track the number of circulating and sequestered iRBCs, containing both dead and alive parasites (Figure 1). The number of circulating parasites were used to determine the rate at which the observed (i.e. circulating) number of iRBC declined post-treatment: These metrics are often measured in clinical trials, including those of Kremsner et al. [5, 16] and allowed us to check our simulation against clinical data. The number of sequestered iRBCs were tracked until they are cleared from the host;

The number of circulating iRBCs containing either living or dead parasites at time *t* is represented as *Q*(*t*) which can be calculated as

 Equation S13

where *x*(*t*) is the number of living parasites in circulating iRBC at time *t*, *Y*(*i*) is the number of parasites killed while in circulating iRBCs during time period *i* post treatment, and *c* was the rate of ‘splenic’ clearance of circulating iRBCs containing dead parasites (by the spleen and other possible host mechanisms such as “pitting”). The clearance half-live, *u*, and its equivalent rate, *c*, are interconverted using the formula

$u=\frac{ln\left( 2 \right)}{c}$ Equation S14

The parasite reduction ratio (PRR) is simply the ratio of iRBC circulating at time of treatment to the number circulating at a given time of follow-up *Q(t)*. PRR is usually measured over 48 hours (PRR_48_) but we later check our model against the clinical observations of Kremsner *et al.* [5] who measured PRR over 24 hours (PRR_24_).

# Simulating treatment of adults

Most malaria mortality occurs in children under 5 years old (and pregnant women) in areas of intense falciparum transmission in sub-Saharan Africa [17]. However, severe malaria does occur in adults in areas of low transmission, where patients have low levels of acquired immunity. The PK parameters from Hendriksen *et al.* we use in our simulations were obtained from children [8], as were the PK parameters from Kremsner *et al.* [5]. We therefore wanted to adapt our model to replicate treatment in adults with the longer durations of artesunate killing that would be expected. We produced a distribution of ‘adult’ durations of artesunate killing by increasing the duration obtained from each set of paediatric PK values by 50%, based on the assumption that

We can use data from Zaloumis *et al.* [18] to calculate artesunate clearance as:

$$Cl= \frac{Dose *F}{AUC}$$

Using their DHA exposure figure of 2,077 h *ng/ml and assuming that a 60kg adult has an exposure of 2800 h *ng/ml, noting that *F* and dose are equal across groups, the ratio of CL of children: adults can be calculated as 1.35. This is not as large as our assumption of 50% increase. However, combined with our results (below), the larger duration serves to illustrate that there is little difference in the ratios of our outcome metrics between children and adults.

This produced the distribution shown in **S2 Figure.** Typical values of this distribution are around 4 to 6 hours artesunate killing, consistent with expectations in adults. Using this distribution in our simulations produced percentages of patients with PRR_24_>99% of 75% and 72% for the five-dose i.m. and the three-dose i.m. regimen, respectively (S3 Table). As would be expected, a longer duration of artesunate killing leads to higher PRR_24_.

We can compare the ratios of our outcome metrics for standard v simplified regimen and sensitive v resistant parasites across the paediatric population with the median ratios obtained using this longer duration of artesunate killing (**S4 Table**). Crucially, there is little difference in the values.

In short, the “adult” treatments modelled by increasing duration of artesunate killing closely match those obtained for the paediatric formulation described in the main text when comparing the impact of regimen change and resistance, and we are reasonably confident that our model is appropriate for investigations of adult patients with severe malaria

# Metrics of severe malaria pathology

As far as we are aware, there is no biological proxy, or direct clinical measurement, for the rate of detachment of iRBCs containing dead parasites in vivo, and it is unknown whether all sequestered iRBCs cause the same pathological burden irrespective of how long they have been sequestered. We therefore use two simple metrics of pathology to enable us to compare the impact of different drug regimens and resistance. Note that we are not attempting the definitive description of severe malaria pathology but present a flexible methodology that allows users to construct more sophisticated models of pathology should they so wish. In the main text we assumed a simple exponential decline of pathology quantified as the pathological recovery rate *r* and that it applies to all sequestered iRBCs containing dead parasites, but we envisage this being expanded in a number of ways, most likely:

- Pathology may be affected by duration of binding e.g. a iRBC that has been sequestered for three hours may have caused less damage than one sequestered for 30 hours; it would be possible to construct a pathology index that reflects this (although this would entail constructing an index to quantify this relationship between duration and pathology).
- The reduction in pathology may not be linear. For example, it may decline very slowly for the first few hours following parasite death, then very rapidly thereafter (or vice versa).
- We assumed pathology was proportional to the number of sequestered parasites and their lingering pathology. Readers may prefer to set a pathological trigger (e.g. >10^9^ sequestered parasites) and use time above that limit as a metric for pathology.

In short there is great flexibility to model pathology, but there is little precedent on how to properly construct or calibrate a definitive model. Hence, we chose the simple methodology described in the main text to minimise complexity and arbitrary choice of parameters and to avoid “cherry picking” metrics to support our results.

Severe malaria is the result of a myriad symptoms and patients often present with different levels of cerebral malaria, severe anaemia, metabolic derangement and respiratory distress. We assumed that reducing sequestered parasitaemia is the key therapeutic objective and that the MPL and AUC_PL_ are realistic metrics to compare treatment regimens of severe malaria. ­We would stress that these metrics do not have to reflect pathology exactly, but are sufficiently accurate proxies that they can serve as comparisons for the clinical efficacy of treatment regimens. The simplifications in our model calibrations primarily reflect the currently poor understanding of the pathology of severe malaria [19], however, one advantage of our methodology is that it is highly flexible and transparent. Users may easily change the calibration to reflect their beliefs of the underlying pathology and test how treatment regimens impact prognosis. For example, respiratory distress syndrome is associated with higher levels of circulating parasitaemia and the clinical priority may be to reduce circulating parasitaemia [20], in which case more detailed analysis of apparent PRR could be conducted [21].

We focus in this manuscript on pathology in the first 24 and 48 hour periods. Pathological effects are known to last much longer and, for example, neurological sequalae can be observed months after treatment[22]. We conjecture that this long-lasting pathology is not due to parasites present at the time of observations but are long-lasting consequences of the parasitaemia and pathology that occurred within the first 48 hours as the pathological load has typically fallen by 4 orders of magnitudes by 48 hours. We therefore believe that our results apply equally well to these longer lasting pathological effects as to the more immediate metrics of death within 24 or 48 hours.

We observe little difference between ratios of AUC_PL_ and MPL when comparing regimens or resistance (i.e., the ratios of AUC_PL_ are close to the ratios of MPL). This occurs because, for each time period, the highest point of pathological load is almost always the earliest hour (i.e., highest pathological load is at 0 hours in 0-12h, 12 hours in 12-24h, so on). Consequently, the MPL for each patient in a time period typically occurs at the very beginning of that time period. It follows that the largest contributions to AUC_PL_ also occur at the very beginning of the time period; Figure 1 (panel B) shows that pathological load follows a linear decline over time.

The similarity between ratios of AUC_Pl_ and MPL is thus un-concerning, it follows that a patient with a higher MPL will have a higher AUC_Pl_ and any parameters that affect one will affect the other equally (i.e., initial parasite number, mean age-bin).

# Impact of the recovery rate *r* (and its half-life)

The key unknown parameter in our simulations is the pathological recovery rate *r.* We vary its value in our simulations by altering its half-life, i.e. the amount of time it takes pathology from sequestered parasites to reduce by half, following the death of the parasite. There are no clinical estimates of this parameter, so we arbitrarily vary it between 4 and 12 hours in our simulations. Most death occurs within 24 hours of admission, so we selected a 12-hour half-life as the upper limit because, if pathology lingered longer, then we would presumably expect more deaths in the post-24hour period. Four hours was taken as the minimum because under this assumption 50% of the pathology disappears by 4 hours, 75% by 8 hours, 87.5% by 12 hours, and this seems rapid. Future researchers can change these values as they see fit or contact us and we can re-run simulations to their specifications. The key operational question is to what extent our assumptions of value of *r* alter the results presented in the main manuscript. We show below that there is negligible impact on our two key comparisons i.e. the impact of alternative regimens and the impact of resistance

*Impact of pathological recovery rate on the baseline scenario i.e. treatment of artemisinin-sensitive parasites with the standard, WHO-recommended regimen*.

We show the impact of half-life on the values of AUC_PL_ and MPL (for patients with sensitive parasites treated under the standard regimen) (S4 Figure); a shorter half-life (indicating a faster rate of clearance) results in lower outcome metrics. This is unsurprising – a faster resolution of pathology from dead sequestered parasites would result in better outcomes. The baseline was conducted purely to establish basic dynamics of treatment and pathology and to check the results were consistent with our expectations. This is clearly the case i.e. that increased recovery rate causes increased pathology.

*Impact of pathological recovery rate on the impact of artemisinin resistance*.

The assumed value of pathological recovery rate had no effect on our estimates of the impact of artemisinin resistance (Figure 6, main text; S10 Table; S5 Figure).

*Impact of pathological recovery rate on the relative performance of attentive regimens.*

The assumed value of pathological recovery rate had a negligible impact when comparing the relative performance of simplified versus standard regimen in the 0-12, 12-24 and 0-24h time periods (Figure 4, main text; S8 Table). However, at 24-48h there is notable negative correlation between AUC_PL_ and MPL and half-life of *r* meaning that the simplified regimen performs increasingly worse during this period as pathological recovery rate increases (i.e. half-life decreases) (S6 Figure).

The most plausible reason for the pattern can be explained by recalling that pathological load is the sum of two contributions: the pathology caused by alive sequestered parasites (first term of Equation 1, main text) and the lingering pathology due to previously-killed parasites (the second term of Equation 1, main text). The pathological recovery rate only affects the second term and so alters the relative size of the two contributions to the overall pathological load. This effect will be explained intuitively and illustrated by dynamics of treatment in an exemplar patient shown on S3 Figure, (note that as patient parameters vary in our simulation, individual patients will have different plots, this one is illustrative). We first discuss the first term of Equation 1 (main text) i.e. number of alive sequestered parasites. The two doses of artemisinin in the first day means that the WHO-standard regimen kills more parasites (for reasons more fully explained in [23]); so the alive, sequestered component is always higher in the simplified than standard treatment (S3 Figure Panel A v B and Panels C vs D). However, the lingering post-mortem pathology from previously killed parasites (the second term in equation 1 (main text)) obscures this difference in live parasites. In the first 24 hours post treatment, the lingering pathology of killed parasites is large, more than an order of magnitude higher than live sequestered burden (S3 Figure), and there is negligible difference between the regimens. After 24 hours the lingering pathology is not such a dominant component, the effect of live sequestered parasites starts to become noticeable (S3 Figure). Increased values of pathological recovery rate (lower half-life) means the lingering pathology becomes a smaller contribution to pathological load and the difference between the regimens becomes more apparent; hence after around 24 hours, the value of pathological recovery does start to have an impact on the comparison between the simplified and standard regimen. To make this algebra clearer, recall that we are dealing with ratios:

$$Ratio of outcome metric=\frac{alive sequestered parasites \left( simplified regimen \right)+lingering pathology (simplified regimen)}{alive sequestered parasites \left( standard regimen \right)+lingering pathology (standard regimen)}$$

(see also equation 1, main text)

Lingering pathology is extremely large in the first 24 hours due to the large amount of killed parasites after initial treatment and makes up an overwhelming proportion of the pathological load. There is negligible difference between the regimens in terms of the lingering pathology (which is primarily governed by the recovery rate, *r*), and so because the lingering pathology component of the equation is by far the largest, the ratio of the outcome metrics will be very close to 1. However, the magnitude of lingering pathology values falls over time post treatment (due to it being resolved by the rate of recovery, *r*) and is lower in the 24-48h period than in 0-24h. These lower values are less able to obscure the difference in effectiveness between the regimens in terms of the number of alive sequestered parasites, so the ratios increase and become more variable (S5 Figure). This can be observed in S3 Figure: Compare panels A and B to panels C and D – with the faster resolution of pathology afforded by a shorter half-life (A, B), the alive sequestered parasites constitute the largest proportion of pathological load by hour 48. With a longer half-life (C, D), the lingering pathology is still the largest component. The faster pathology is resolved, the smaller the magnitude of lingering pathology and the larger the value of the ratio.

Using illustrative values, at 24h when lingering pathology is high:

$$ratio=\frac{1e8 \left( alive sequestered \right)+1e10 (lingering pathology)}{1e7 (alive sequestered)+ 1e10 (lingering pathology)}=1.009$$

At 48h, when lingering pathology is low:

$$ratio=\frac{1e8 \left( alive sequestered \right)+1e8 (lingering pathology)}{1e7 (alive sequestered)+ 1e8 (lingering pathology)}=1.81$$

In summary, pathological recovery rate has no impact in the first 24 hours but has an important impact on the relative performance of the regimens (quantified as ratios of AUC_PL_) in the later, 24-48h post-treatment period (see **S8 Table** & **S17 Figure** ) (note that it is not important for MPL as the value of MPL in the 24-48h period typically occurs at 24 hours, this is reflected in the PRCC analysis showing no correlation between MPL at 24-48h and the half-life of *r*). We believe our analysis of different drug regimens to treat severe malaria is robust to assumptions of the value of recovery rate for the following two reasons. Firstly, in the critical first 24 hour period following treatment (the period focused on by Kremsner et al. [5]), PRCC results indicate that the half-life parameter has no impact on the ratio of AUC_PL_ and MPL of the regimens. This is further evidenced by **S3 Figure** and **S17 Figure –** even with an extremely short half-life, the pathological load did not differ sufficiently in the first 24 hours to make any difference between the regimens when *r* changes. Secondly, pathological load is much higher in the 0-24h period than the 24-48h period, so the lack of impact of *r* in the 0-24h period is critical (AUC_PL_ and MPL at 24-48h are generally 20-30% of the values at 0-24h, though this will vary depending on the value of patient parameters, including the half-life of the pathological recovery rate) This reflects our belief that pathology/death in the 24-48 hour period post-treatment is not described solely by pathological metrics in that period: rather it is the cumulated damage since treatment was initiated that is important and that includes the metrics in the 0-24h period. Finally, it is clear from our full set of results that while an extremely low half-life is associated with better outcome from the standard regimen in all time periods except 0-12h, the standard regimen produces lower AUC_PL_ across the entire range of parameter values (MPL is equivalent at 0-24h and 12-24h), so no matter the parameterization of the half-life of *r*, the standard regimen still produces lower outcome metrics

# Choice of Standard Deviation (SD) of the initial mean age bin and modelling uniform age bin distributions.

We constrained the SD of the mean age-bin between 2 and 4. This results in reasonably narrow age-bin distributions and reflects existing belief and observations that severe malaria infections are synchronized [12, 24].

Mathemtically, 95% of the population fall with +/- 1.96 SD of the mean. Our maximum of SD=4 therefore means that 95% of parasites fall within a 16 hour develolment perod which is one third of the 48 hour intraerythrocyte developmental cycle This is consistent with clinical observations that some clones are genetically unobservable one day (i.e, sequestered) and observable (i.e, circulating) the next day (see [24] for a complete discussion of the synchronization of parasites and note that in this model, 100% of parasites are circulating hours 1-11 then gradually sequester, see elsewhere in this Supplementary Information and [3]).

Any value of SD can be modelled. The key change as the distribution widens is that the mean age-bin parameter becomes less important. [This is intuitive as the importance of this parameter derives from the fact that, with a narrow distribution, patients with certain initial mean age-bins have treatment falling in less/non-sensitive bins (as we discuss extensively in the main text)]. We did also run the simulations with a uniform distribution of age-bins. The technical problem with this distribution is that there is no “mean age bin” so we cannot analyse its impact nor incorporate this distribution into our formal sensitivity analyses (S7 Table-S10 Table); as expected from the explanation immediately above (i.e. “this is intuitive…”) under these circumstances it is fair to claim, as Kremsner et al. did, that the simplified regimen is “non-inferior”. This occurs because when parasites are uniformly distributed there are no “at risk” patients with infections clustered in early mean age-bins at the time of treatment, and all patients will experience high levels of killing from the initial artesunate dose.

# Supplementary information references:

1. Milner DA, Vareta J, Valim C, Montgomery J, Daniels RF, Volkman SK, et al. Human cerebral malaria and Plasmodium falciparum genotypes in Malawi. Malaria Journal. 2012;11(1):35. doi: 10.1186/1475-2875-11-35.

2. Oyedeji SI, Awobode HO, Kun J. Limited Genetic Diversity and Low Multiplicity of Plasmodium falciparum Infections in Children with Severe Malaria in Lafia, North-central Nigeria. Journal of Experimental & Clinical Medicine. 2013;5(4):143-7. doi: <https://doi.org/10.1016/j.jecm.2013.06.014>.

3. Saralamba S, Pan-Ngum W, Maude RJ, Lee SJ, Tarning J, Lindegardh N, et al. Intrahost modeling of artemisinin resistance in Plasmodium falciparum. P Natl Acad Sci USA. 2011;108(1):397-402. doi: DOI 10.1073/pnas.1006113108. PubMed PMID: ISI:000285915000073.

4. World Health Organization. Management of severe malaria2012.

5. Kremsner PG, Adegnika AA, Hounkpatin AB, Zinsou JF, Taylor TE, Chimalizeni Y, et al. Intramuscular Artesunate for Severe Malaria in African Children: A Multicenter Randomized Controlled Trial. PLoS Med. 2016;13(1):e1001938. doi: 10.1371/journal.pmed.1001938.

6. World Health Organization. Guidelines for the treatment of malaria.2015.

7. Hodel EM, Kay K, Hastings IM. Incorporating Stage-Specific Drug Action into Pharmacological Modeling of Antimalarial Drug Treatment. Antimicrobial agents and chemotherapy. 2016;60(5):2747-56. doi: 10.1128/AAC.01172-15. PubMed PMID: PMC4862506.

8. Hendriksen IC, Mtove G, Kent A, Gesase S, Reyburn H, Lemnge MM, et al. Population pharmacokinetics of intramuscular artesunate in African children with severe malaria: implications for a practical dosing regimen. Clinical pharmacology and therapeutics. 2013;93(5):443-50. Epub 2013/03/21. doi: 10.1038/clpt.2013.26. PubMed PMID: 23511715; PubMed Central PMCID: PMCPMC3630454.

9. Hoshen MB, Stein WD, Ginsburg H. Modelling the chloroquine chemotherapy of falciparum malaria: the value of spacing a split dose. Parasitology. 1998;116 ( Pt 5):407-16. Epub 1998/06/06. PubMed PMID: 9614323.

10. Winter K, Hastings IM. Development, evaluation, and application of an in silico model for antimalarial drug treatment and failure. Antimicrob Agents Chemother. 2011;55(7):3380-92. Epub 2011/05/04. doi: 10.1128/aac.01712-10. PubMed PMID: 21537019; PubMed Central PMCID: PMCPmc3122393.

11. Klonis N, Xie SC, McCaw JM, Crespo-Ortiz MP, Zaloumis SG, Simpson JA, et al. Altered temporal response of malaria parasites determines differential sensitivity to artemisinin. Proceedings of the National Academy of Sciences. 2013;110(13):5157-62. doi: 10.1073/pnas.1217452110.

12. Hodel EM, Kay K, Hastings IM. Incorporating Stage-Specific Drug Action into Pharmacological Modeling of Antimalarial Drug Treatment. Antimicrob Agents Chemother. 2016;60(5):2747-56. doi: 10.1128/AAC.01172-15. PubMed PMID: 26902760.

13. Fairhurst RM, Dondorp AM. Artemisinin-Resistant Plasmodium falciparum Malaria. Microbiology spectrum. 2016;4(3). Epub 2016/06/24. doi: 10.1128/microbiolspec.EI10-0013-2016. PubMed PMID: 27337450; PubMed Central PMCID: PMCPMC4992992.

14. Molineaux L, Diebner HH, Eichner M, Collins WE, Jeffery GM, Dietz K. Plasmodium falciparum parasitaemia described by a new mathematical model. Parasitology. 2002;122(4):379-91. Epub 06/18. doi: 10.1017/S0031182001007533.

15. Paget-McNicol S, Gatton M, Hastings I, Saul A. The Plasmodium falciparum var gene switching rate, switching mechanism and patterns of parasite recrudescence described by mathematical modelling. Parasitology. 2002;124(Pt 3):225-35. Epub 2002/04/02. PubMed PMID: 11922425.

16. Kremsner PG, Taylor T, Issifou S, Kombila M, Chimalizeni Y, Kawaza K, et al. A Simplified Intravenous Artesunate Regimen for Severe Malaria. Journal of Infectious Diseases. 2011. doi: 10.1093/infdis/jir724.

17. World Health Organization. World Malaria Report 20162016 13 December 2016. 186 p.

18. Zaloumis SG, Tarning J, Krishna S, Price RN, White NJ, Davis TME, et al. Population Pharmacokinetics of Intravenous Artesunate: A Pooled Analysis of Individual Data From Patients With Severe Malaria. CPT: Pharmacometrics & Systems Pharmacology. 2014;3(11):e145. doi: 10.1038/psp.2014.43. PubMed PMID: PMC4259998.

19. Cunnington AJ, Walther M, Riley EM. Piecing Together the Puzzle of Severe Malaria. Science Translational Medicine. 2013;5(211):211ps18-ps18. doi: 10.1126/scitranslmed.3007432.

20. Cunnington AJ, Riley EM, Walther M. Stuck in a rut? Reconsidering the role of parasite sequestration in severe malaria syndromes. Trends in Parasitology. 2013;29(12):585-92. doi: <http://dx.doi.org/10.1016/j.pt.2013.10.004>.

21. Zaloumis S, Humberstone A, Charman SA, Price RN, Moehrle J, Gamo-Benito J, et al. Assessing the utility of an anti-malarial pharmacokinetic-pharmacodynamic model for aiding drug clinical development. Malaria Journal. 2012;11(1):303. doi: 10.1186/1475-2875-11-303.

22. Moxon CA, Chisala NV, Wassmer SC, Taylor TE, Seydel KB, Molyneux ME, et al. Persistent Endothelial Activation and Inflammation After Plasmodium falciparum Infection in Malawian Children. The Journal of infectious diseases. 2014;209(4):610-5. doi: 10.1093/infdis/jit419.

23. Kay K, Hodel EM, Hastings IM. Altering antimalarial drug regimens may dramatically enhance and restore drug effectiveness. Antimicrobial Agents and Chemotherapy. 2015. doi: 10.1128/aac.00482-15.

24. Färnert A, Lebbad M, Faraja L, Rooth I. Extensive dynamics of Plasmodium falciparum densities, stages and genotyping profiles. Malaria Journal. 2008;7(1):241. doi: 10.1186/1475-2875-7-241.
