# Supplementary material for: Optimal Treatments for Severe Malaria and the Threat Posed by Artemisinin Resistance
Source: J Infect Dis. 2018 Dec 5;219(8):1243–53. doi: 10.1093/infdis/jiy649 (PMC6452316; doi:10.1093/infdis/jiy649)
Supplement: Supplementary Table S1 [file jiy649_suppl_supplementary_table_s1.pdf]

S1 Table: Pharmacokinetic (PK) parameters drawn from Kremsner *et al.* [3]

Parameter	Unit	Abbreviation	Range
Volume of distribution i.m. AS [3]	[L]	$V_{AS,i.m}$	*x*, where mean = 21.1 and CV = 0.97
Volume of distribution i.m. DHA [3]	[L]	$V_{DHA,i.m}$	*x*, where mean = 25.3 and CV = 0.81
Clearance i.m. AS [3]	[L/h]	$CL_{AS,i.m}$	*x*, where mean = 33.3 and CV = 0.81
Clearance i.m. DHA [3]	[L/h]	$CL_{DHA,i.m}$	*x*, where mean = 8.5 and CV = 0.87
