# Supplementary material for: Optimal Treatments for Severe Malaria and the Threat Posed by Artemisinin Resistance
Source: J Infect Dis. 2018 Dec 5;219(8):1243–53. doi: 10.1093/infdis/jiy649 (PMC6452316; doi:10.1093/infdis/jiy649)
Supplement: Supplementary Table S2 [file jiy649_suppl_supplementary_table_s2.pdf]

S2 Table: Pharmacokinetic (PK) parameters drawn from Hendriksen *et al.* [6]

Parameter	Unit	Abbreviation	Range
Volume of distribution i.m. AS [6]	[L]	$V_{AS,i.m}$	*x*, where mean = 28.2 and CV = 0.98
Volume of distribution i.m. DHA [6]	[L]	$V_{DHA,i.m}$	*x*, where mean = 13.5 and CV = 0.98
Clearance i.m. AS (Hendriksen)	[L/h]	$CL_{AS,i.m}$	*x*, where mean = 45.8 and CV = 0.71
Clearance i.m. DHA (Hendriksen)	[L/h]	$CL_{DHA,i.m}$	*x*, where mean = 22.4 and CV = 0.59
Correlation of random effects on V and CL		ηCL/F ARS ~ ηV/F ARS	*X,* where mean = 0.497
