# Supplementary material for: Optimal Treatments for Severe Malaria and the Threat Posed by Artemisinin Resistance
Source: J Infect Dis. 2018 Dec 5;219(8):1243–53. doi: 10.1093/infdis/jiy649 (PMC6452316; doi:10.1093/infdis/jiy649)
Supplement: Supplementary Table S3 [file jiy649_suppl_supplementary_table_s3.pdf]

S3 Table: Clinical outcome (Parasite Reduction Ratio, PRR) observed in simulations using three different pharmacokinetic (PK) parameterizations. (from Kremsner *et al*. [3], Hendriksen *et al*. [6], and Hendriksen *et al.* [6] with increased duration).

Parameter choice	Kremsner et. al [3]		Hendriksen et. al [6]		Hendriksen et. al [6] (50% increase in artesunate duration)	
Regimen	Standard	Simplified	Standard	Simplified	Standard	Simplified
Artesunate duration figure	S1 Figure		Figure 2 (main text)		S2 Figure	
% of patients with >99% PRR at 24h	78%	74%	70%	62%	77%	72%
Population geometric mean PRR at 48h	5.56^{-6}	2.33^{-5}	5.18^{-5}	0.0009	5.21^{-6}	5.28^{-5}
