# Supplementary material for: Optimal Treatments for Severe Malaria and the Threat Posed by Artemisinin Resistance
Source: J Infect Dis. 2018 Dec 5;219(8):1243–53. doi: 10.1093/infdis/jiy649 (PMC6452316; doi:10.1093/infdis/jiy649)
Supplement: Supplementary Table S4 [file jiy649_suppl_supplementary_table_s4.pdf]

S4 Table: Median ratios of AUC_{PL} and MPL across the patient population for 4 time-periods. Parameters are from Hendriksen *et al.* [6] (top) and from Hendriksen *et al.* with 50% increase in artesunate killing duration (bottom, inside []). A ratio of <1 indicates lower metric with the simplified regimen or resistant parasites respectively:

Median ratios for standard v simplified regimen with sensitive parasites				
Time (h)	0-12	0-24	12-24	24-48
AUC	0.99<br>[0.99]	1.03<br>[1.007]	1.12<br>[1.03]	1.49<br>[1.18]
MPL	1<br>[1]	1<br>[1]	0.98<br>[0.98]	1.45<br>[1.17]
Median ratios for sensitive v resistant parasites, treated with standard regimen				
Time (h)	0-12	0-24	12-24	24-48
AUC	1.000003<br>[1.000003]	1.000203<br>[1.000161]	1.000615<br>[1.000505]	1.006516<br>[1.00208]
MPL	1<br>[1]	1<br>[1]	1.000092<br>[1.000081]	1.002546<br>[1.001053]
