# Supplementary material for: Optimal Treatments for Severe Malaria and the Threat Posed by Artemisinin Resistance
Source: J Infect Dis. 2018 Dec 5;219(8):1243–53. doi: 10.1093/infdis/jiy649 (PMC6452316; doi:10.1093/infdis/jiy649)
Supplement: Supplementary Table S6 [file jiy649_suppl_supplementary_table_s6.pdf]

S6 Table: Grouping of the mean age-bin parameter for resistant parasites into ordinal categories according to the amount of killing that occurs in that age-bin, suitable for partial rank correlation coefficient (PRCC) analysis

Age bin	Rank	Killing in age bins
45-5	1	None (i.e. 0*V_{max})
6-17	2	0.1*V_{max}
18-44	3	1*V_{max}
