# Supplementary material for: Optimal Treatments for Severe Malaria and the Threat Posed by Artemisinin Resistance
Source: J Infect Dis. 2018 Dec 5;219(8):1243–53. doi: 10.1093/infdis/jiy649 (PMC6452316; doi:10.1093/infdis/jiy649)
Supplement: Supplementary Table S7 [file jiy649_suppl_supplementary_table_s7.pdf]

S7 Table: PRCC values with corresponding *p* values (brackets) for values of AUC_{PL} and MPL for a patient population simulated with sensitive parasites and treated with the standard regimen, using seven key model parameters.

Outcome Metric	Time period	Parameter						
		Initial parasite number	Initial mean age-bin	Standard deviation	PMR	V_{max}	Half-life of *r*	Artesunate duration
AUC_{PL}	0-12h	0.95 (<0.001)	0.05 (<0.001)	0.001 (0.71)	-0.012 (0.003)	-0.02 (<0.001)	0.05 (<0.001)	-0.004 (0.27)
	0-24h	0.97 (<0.001)	-0.01 (<0.001)	-0.001 (0.77)	-0.003 (0.386)	-0.02 (<0.001)	0.11 (<0.001)	-0.02 (<0.001)
	12-24h	0.95 (<0.001)	-0.14 (<0.001)	0.002 (0.52)	0.003 (0.34)	-0.02 (<0.001)	0.22 (<0.001)	-0.04(<0.001)
	24-48h	0.83 (<0.001)	-0.09 (<0.001)	0.015 (<0.001)	0.015 (<0.001)	-0.02 (<0.001)	0.46 (<0.001)	-0.04 (<0.001)
MPL	0-12h	0.97 (<0.001)	0.08 (<0.001)	-0.02 (<0.001)	-0.013 (<0.001)	-0.02 (<0.001)	0.008 (0.03)	-0.005 (0.17)
	0-24h	0.97 (<0.001)	0.09 (<0.001)	-0.02 (<0.001)	-0.007 (0.06)	-0.02 (<0.001)	0.01 (0.01)	-0.01 (0.01)
	12-24h	0.096 (<0.001)	-0.14 (<0.001)	-0.005 (0.21)	0.003 (0.47)	-0.03 (<0.001)	0.14 (<0.001)	-0.03 (<0.001)
	24-48h	0.89 (<0.001)	-0.1 (<0.001)	0.02 (<0.001)	0.01 (0.001)	-0.02 (<0.001)	0.35 (<0.001)	-0.04 (<0.001)

PRCC: Partial Rank Correlation Coefficient, AUC_{PL}: Area under the pathological load curve, MPL: Maximum value of pathological load, PMR: Parasite multiplication rate, V_{max} : Maximal rate of artesunate killing, *r*: pathological load recovery rate.
