# Supplementary material for: Optimal Treatments for Severe Malaria and the Threat Posed by Artemisinin Resistance
Source: J Infect Dis. 2018 Dec 5;219(8):1243–53. doi: 10.1093/infdis/jiy649 (PMC6452316; doi:10.1093/infdis/jiy649)
Supplement: Supplementary Table S8 [file jiy649_suppl_supplementary_table_s8.pdf]

S8 Table: PRCC values with corresponding *p* values (brackets) for ratios of AUC_{PL} and MPL for the simplified v standard regimen for a patient population simulated with sensitive parasites using seven key model parameters. The ratio is calculated as $\frac{Outcome\ metric\ of\ simplified\ regimen}{Outcome\ metric\ of\ standard\ regimen}$ such that higher ratios (and thus, positive correlation) indicate better performance of the standard regimen.

Outcome Metric	Time period	Parameter						
		Initial parasite number	Initial mean age-bin	Standard deviation	PMR	V_{max}	Half-life of *r*	Artesunate duration
AUC_{PL}	0-12h	0.007 (0.07)	0.64 (<0.001)	-0.02 (<0.001)	-0.006 (0.1)	0.02 (<0.001)	0.13 (<0.001)	0.24 (<0.001)
	0-24h	-0.003 (0.41)	-0.63 (<0.001)	0.09 (<0.001)	0.013 (<0.001)	0.007 (0.06)	-0.16 (<0.001)	0.009 (0.02)
	12-24h	-0.003 (0.35)	-0.64 (<0.001)	0.1 (<0.001)	0.012 (0.002)	-0.006 (0.1)	-0.28 (<0.001)	-0.03 (<0.001)
	24-48h	-0.005 (0.24)	-0.66 (<0.001)	-0.08 (<0.001)	0.04 (<0.001)	-0.04 (<0.001)	-0.38 (<0.001)	-0.09 (<0.001)
MPL	0-12h	-0.009 (0.02)	0.59 (<0.001)	0.003 (0.4)	-0.00005 (0.89)	-0.02 (<0.001)	-0.003 (0.52)	0.1 (<0.001)
	0-24h	0.002 (0.58)	0.55 (<0.001)	0.11 (<0.001)	0.06 (<0.001)	0.002 (0.52)	-0.02 (<0.001)	0.03 (<0.001)
	12-24h	0.01 (0.002)	0.62 (<0.001)	0.06 (<0.001)	0.03 (<0.001)	0.01 (<0.001)	0.22 (<0.001)	0.14 (<0.001)
	24-48h	-0.006 (0.15)	-0.67 (<0.001)	0.08 (<0.001)	0.007 (0.08)	-0.008 (0.04)	-0.33 (<0.001)	-0.04 (<0.001)

PRCC: Partial Rank Correlation Coefficient, AUC_{PL}: Area under the pathological load curve, MPL: Maximum value of pathological load, PMR: Parasite multiplication rate, V_{max} : Maximal rate of artesunate killing, *r*: pathological load recovery rate.
