# Supplementary material for: Optimal Treatments for Severe Malaria and the Threat Posed by Artemisinin Resistance
Source: J Infect Dis. 2018 Dec 5;219(8):1243–53. doi: 10.1093/infdis/jiy649 (PMC6452316; doi:10.1093/infdis/jiy649)
Supplement: Supplementary Table S9 [file jiy649_suppl_supplementary_table_s9.pdf]

S9 Table: PRCC values with corresponding *p* values (brackets) for ratios of AUC_{PL} and MPL for the simplified v standard regimen for a patient population simulated with resistant parasites using seven key model parameters. The ratio is calculated as $\frac{Outcome\ metric\ of\ simplified\ regimen}{Outcome\ metric\ of\ standard\ regimen}$ such that higher ratios (and thus, positive correlation) indicate better performance of the standard regimen.

Outcome Metric	Time period	Parameter						
		Initial parasite number	Initial mean age-bin	Standard deviation	PMR	V_{max}	Half-life of *r*	Artesunate duration
AUC_{PL}	0-12h	0.007 (0.05)	0.63 (0)	-0.02 (<0.001)	-0.07 (0.08)	0.019 (<0.001)	0.13 (<0.001)	0.23 (0)
	0-24h	-0.007 (0.08)	-0.57 (0)	0.11 (<0.001)	0.03 (<0.001)	0.01 (<0.001)	-0.15 (2.22)	0.02 (<0.001)
	12-24h	-0.008 (0.04)	-0.6 (0)	0.11 (<0.001)	0.03 s(<0.001)	-3.45^{-5} (0.99)	-0.28 (0)	-0.01 (<0.001)
	24-48h	-0.006 (0.11)	-0.64 (0)	0.07 (<0.001)	0.05 (<0.001)	-0.03 (<0.001)	-0.4 (0)	-0.09 (<0.001)
MPL	0-12h	-0.002 (0.48)	0.54 (0)	-0.008 (0.03)	-0.002 (0.47)	-0.02 (<0.001)	0.001 (0.73)	0.08 (<0.001)
	0-24h	-0.01 (0.002)	0.31 (0)	0.16 (0)	0.04 (<0.001)	0.003 (0.44)	-0.01 (<0.001)	0.05 (<0.001)
	12-24h	0.002 (0.47)	0.41 (0)	0.1 (<0.001)	0.015 (<0.001)	0.027 (<0.001)	0.19 (0)	0.15 (0)
	24-48h	-0.007 (0.06)	-0.66 (0)	0.085 (<0.001)	0.017 (<0.001)	0.003 (0.41)	-0.33 (0)	-0.01 (<0.001)

PRCC: Partial Rank Correlation Coefficient, AUC_{PL}: Area under the pathological load curve, MPL: Maximum value of pathological load, PMR: Parasite multiplication rate, V_{max} : Maximal rate of artesunate killing, *r*: pathological load recovery rate.
