# Supplementary material for: Optimal Treatments for Severe Malaria and the Threat Posed by Artemisinin Resistance
Source: J Infect Dis. 2018 Dec 5;219(8):1243–53. doi: 10.1093/infdis/jiy649 (PMC6452316; doi:10.1093/infdis/jiy649)
Supplement: Supplementary Table S10 [file jiy649_suppl_supplementary_table_s10.pdf]

S10 Table: PRCC values with corresponding *p* values (brackets) for **ratios** of AUC_{PL} and MPL for resistant v sensitive parasites for a patient population simulated treated with the standard regimen, using seven key model parameters. The ratio is calculated as $\frac{Outcome\ metric\ of\ resistant\ parasites}{Outcome\ metric\ of\ sensitive\ parasites}$ such that higher ratios (and thus, positive correlation) indicate better outcomes with sensitive parasites

Outcome Metric	Time period	Parameter						
		Initial parasite number	Initial mean age-bin	Standard deviation	PMR	V_{max}	Half-life of *r*	Artesunate duration
AUC_{PL}	0-12h	0.003 (0.51)	-0.55 (<0.001)	0.18 (<0.001)	0.004 (0.32)	0.009 (0.03)	-0.005 (0.18)	-0.01 (<0.001)
	0-24h	0.009 (0.03)	-0.42 (<0.001)	0.29 (<0.001)	0.025 (<0.001)	-0.005 (0.15)	-0.02 (<0.001)	-0.03 (<0.001)
	12-24h	0.01 (0.02)	-0.4 (<0.001)	0.3 (<0.001)	0.24 (<0.001)	-0.006 (0.15)	-0.04 (<0.001)	-0.02 (<0.001)
	24-48h	0.01 (0.001)	-0.19 (<0.001)	0.31 (<0.001)	0.06 (<0.001)	-0.07 (<0.001)	-0.13 (<0.001)	-0.11 (<0.001)
MPL	0-12h	0.003 (0.36)	-0.4 (<0.001)	0.05 (<0.001)	-0.0007 (0.87)	-0.001 (0.71)	0.01 (0.02)	-0.05 (<0.001)
	0-24h	0.002 (0.52)	-0.35 (<0.001)	0.06 (<0.001)	0.02 (<0.001)	-0.0001 (0.97)	0.02 (<0.001)	-0.05 (<0.001)
	12-24h	0.004 (0.3)	-0.51 (<0.001)	0.21 (<0.001)	0.007 (<0.001)	0.01 (0.02)	-0.01 (0.04)	-0.0002 (0.96)
	24-48h	0.01 (0.002)	-0.33 (<0.001)	0.32 (<0.001)	0.03 (<0.001)	-0.01 (<0.001)	-0.1 (<0.001)	-0.05 (<0.001)

PRCC: Partial Rank Correlation Coefficient, AUC_{PL}: Area under the pathological load curve, MPL: Maximum value of pathological load, PMR: Parasite multiplication rate, V_{max} : Maximal rate of artesunate killing, *r*: pathological load recovery rate.
