# Supplementary material for: Optimal Treatments for Severe Malaria and the Threat Posed by Artemisinin Resistance
Source: J Infect Dis. 2018 Dec 5;219(8):1243–53. doi: 10.1093/infdis/jiy649 (PMC6452316; doi:10.1093/infdis/jiy649)

S1 Figure: Distribution of artemisinin killing duration obtained when using the pharmacokinetic (PK) parameters reported in Kremsner *et. al* [3]


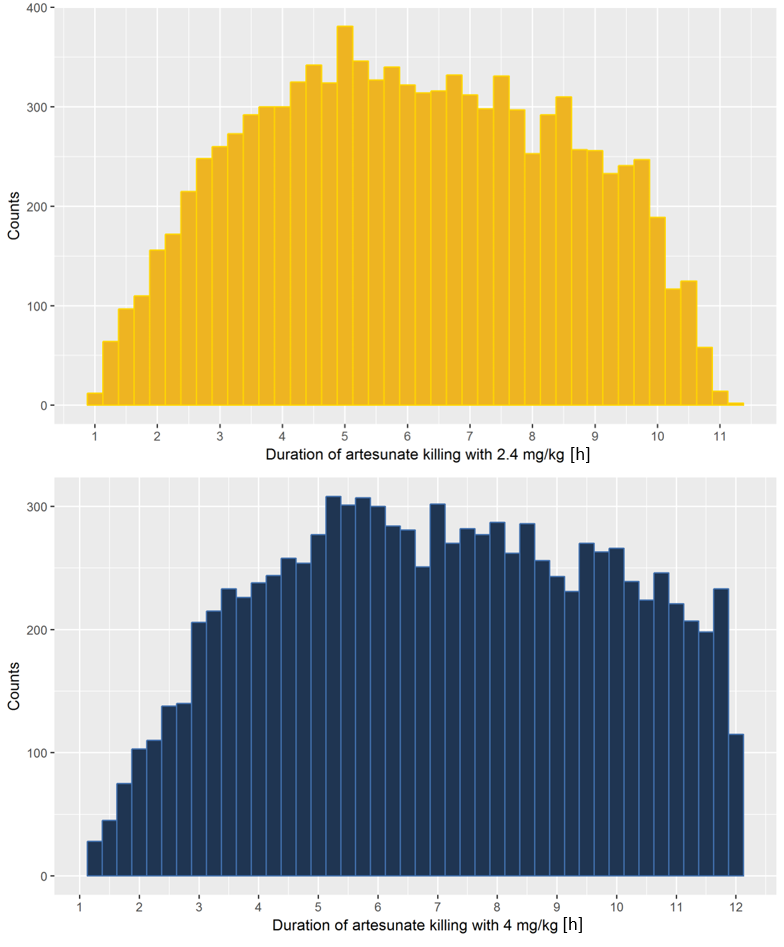

Supplement: Supplementary Figure S1 [file jiy649_suppl_supplementary_figure_s1.docx]
