# Supplementary material for: Optimal Treatments for Severe Malaria and the Threat Posed by Artemisinin Resistance
Source: J Infect Dis. 2018 Dec 5;219(8):1243–53. doi: 10.1093/infdis/jiy649 (PMC6452316; doi:10.1093/infdis/jiy649)

S3 Figure: Plots to illustrate the proportion of total pathological load contributed by alive sequestered parasites and by lingering post-mortem pathology, and the differences in these contributions when the half-life of the recovery rate *r* is varied. With a lower half-life (faster recovery), alive sequestered parasites constitute a larger proportion of pathological load and so differences between standard and simplified regimens with respect to the number of alive, sequestered parasites become more apparent with the assumption of faster recover.

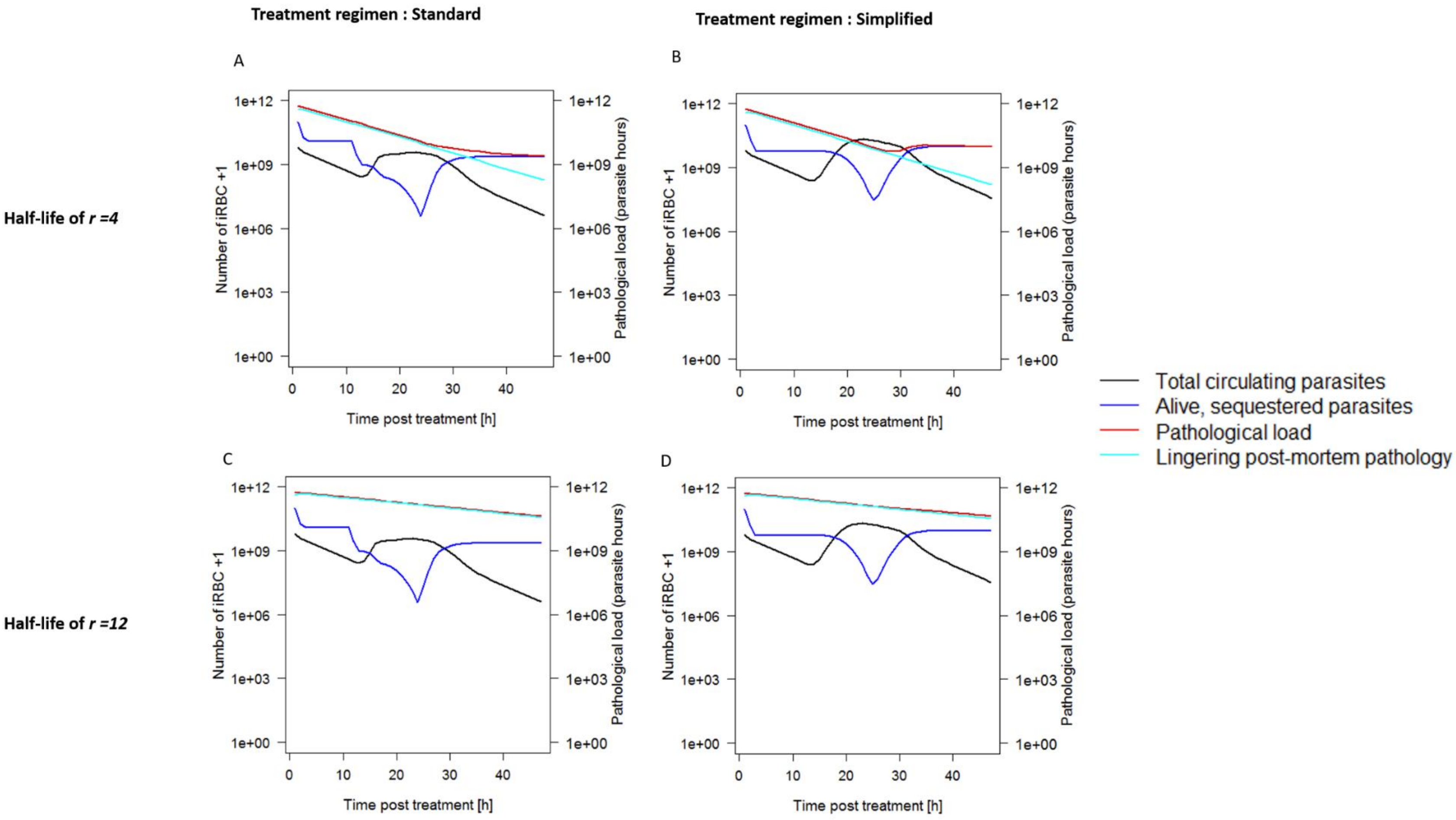

Supplement: Supplementary Figure S3 [file jiy649_suppl_supplementary_figure_s3.pdf]
