# Supplementary material for: Optimal Treatments for Severe Malaria and the Threat Posed by Artemisinin Resistance
Source: J Infect Dis. 2018 Dec 5;219(8):1243–53. doi: 10.1093/infdis/jiy649 (PMC6452316; doi:10.1093/infdis/jiy649)

S4 Figure: Population values of $AUC_{PL}$ and MPL for patients with sensitive parasites treated with the standard regimen, plotted according to half-life of the pathological load recovery rate *r* across four time periods post-treatment: 0-12h, 0-24h, 12-24h and 24-48h.

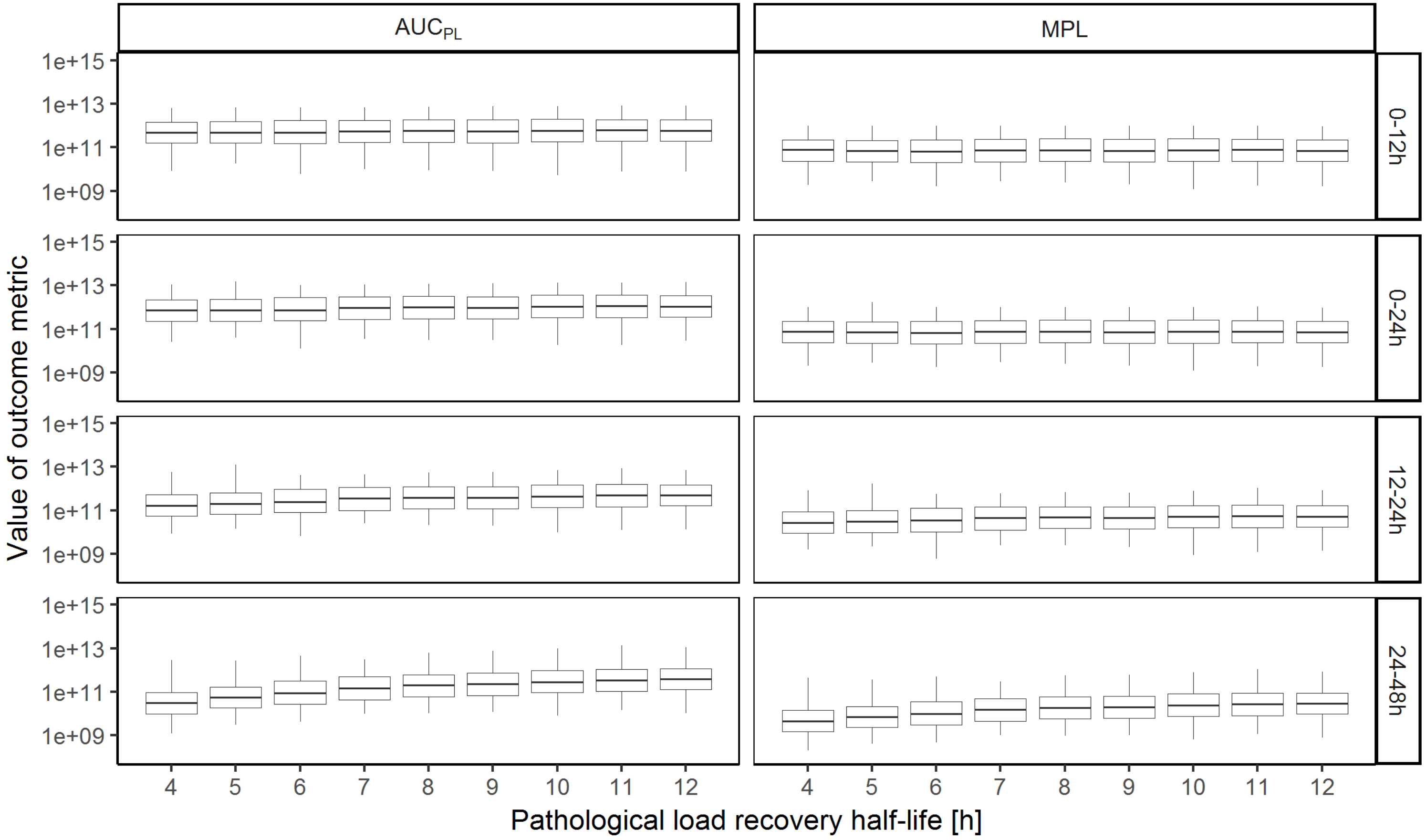

Supplement: Supplementary Figure S4 [file jiy649_suppl_supplementary_figure_s4.pdf]
