# Supplementary material for: Optimal Treatments for Severe Malaria and the Threat Posed by Artemisinin Resistance
Source: J Infect Dis. 2018 Dec 5;219(8):1243–53. doi: 10.1093/infdis/jiy649 (PMC6452316; doi:10.1093/infdis/jiy649)

S7 Figure: PRCC analysis using Spearman's Rho of model parameters on the ratios of $AUC_{PL}$ and MPL for the standard v the simplified regimen when parasites are resistant. A PRCC of over 0.3 (+ or -) indicates that the parameter has notable correlation with the ratio.

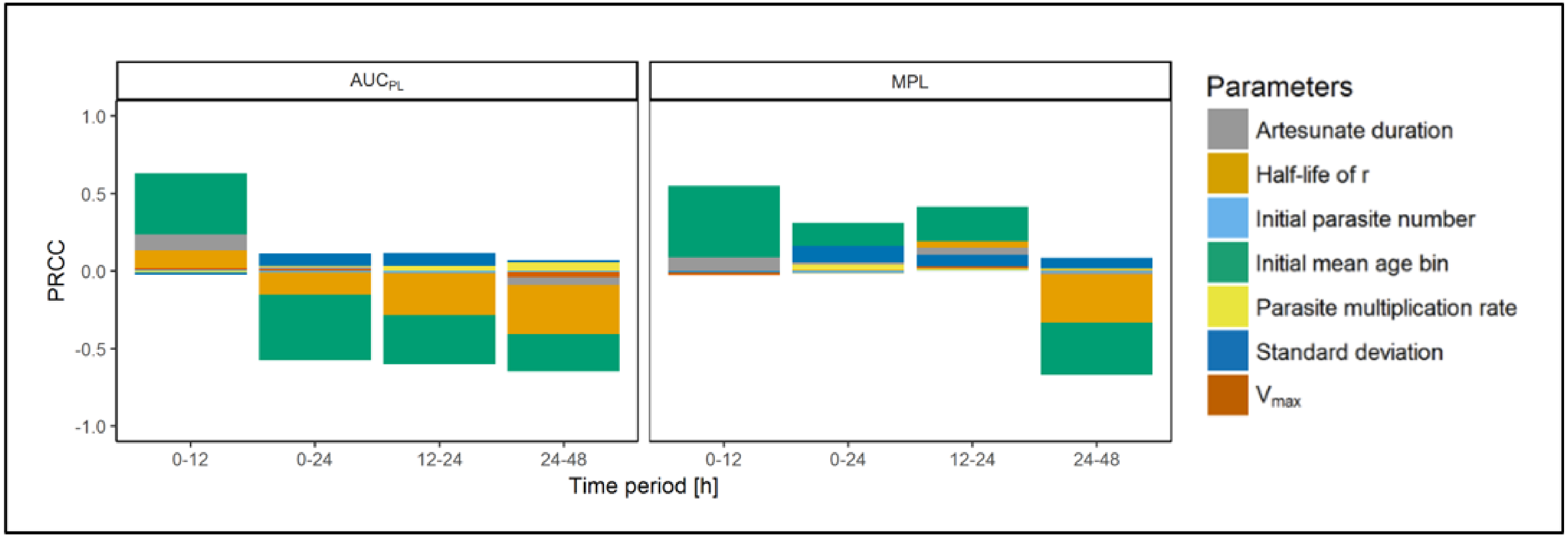

Supplement: Supplementary Figure S7 [file jiy649_suppl_supplementary_figure_s7.pdf]
